# Cancer‐associated fibroblasts secrete Wnt2 to promote cancer progression in colorectal cancer

**DOI:** 10.1002/cam4.2523

**Published:** 2019-08-29

**Authors:** Takashi Aizawa, Hideaki Karasawa, Ryo Funayama, Matsuyuki Shirota, Takashi Suzuki, Shimpei Maeda, Hideyuki Suzuki, Akihiro Yamamura, Takeshi Naitoh, Keiko Nakayama, Michiaki Unno

**Affiliations:** ^1^ Department of Surgery Tohoku University Graduate School of Medicine Sendai Japan; ^2^ Department of Cell Proliferation ART Tohoku University Graduate School of Medicine Sendai Japan; ^3^ Division of Interdisciplinary Medical Science ART Tohoku University Graduate School of Medicine Sendai Japan; ^4^ Department of Pathology and Histotechnology Tohoku University Graduate School of Medicine Sendai Japan

**Keywords:** cancer‐associated fibroblast, colorectal cancer, gene set enrichment analysis, RNA sequencing, Wnt2

## Abstract

Recent studies have shown that the tumor microenvironment plays a significant role in the progression of solid tumors. As an abundant component of the tumor microenvironment, cancer‐associated fibroblasts (CAFs) have been shown to promote tumorigenesis and cancer aggressiveness, but their molecular characteristics remain poorly understood. In the present study, paired CAFs and normal fibroblasts (NFs) were isolated from five colorectal cancer (CRC) tissues from patients who underwent surgical resection. The gene expression profiles of CAFs and NFs identified by RNA sequencing were compared to understand the complex role of CAFs in cancer progression. Gene Set Enrichment Analysis revealed that the gene sets related to the Wnt signaling pathway were highly enriched in CAFs, as well as TGFβ signaling, which is considered to be a regulator of CAFs. Among the components of this pathway, Wnt2 was specifically expressed. The observations led us to speculate that Wnt2 is extremely involved in regulating CRC progression by CAFs. Thus, we performed immunohistochemical analysis on Wnt2 in 171 patients who underwent surgery for colorectal adenocarcinoma. Positive staining for Wnt2 was mainly observed in cancer stroma, although the immunoreactivity was weak in cancer cells. Wnt2 expression in CAFs was significantly correlated with depth of tumor (*P* < .001), lymph node metastasis (*P* = .044), TNM stage (*P* = .010), venous invasion (*P* < .001), and recurrence (*P* = .013). Subsequent in vitro analyses were conducted using conditioned medium (CM) from immortalized CAFs transfected with siRNA targeting Wnt2. As a result, cancer cell invasion and migration were significantly decreased in the CM from immortalized CAFs transfected with siRNA targeting Wnt2. Our findings indicated that Wnt2 protein released from CAFs enhances CRC cell invasion and migration. In conclusion, Wnt2 secreted by CAFs plays a key role in cancer progression and is a potential therapeutic target for CRC.

## Graphical abstract

The analysis here involved comparing gene expression between cancer associated and normal fibroblasts using RNA sequencing, which identified that components of the Wnt signaling pathway were highly expressed in cancer associated fibroblasts, and Wnt2 in particular. We then conducted immunohistochemical analysis on Wnt2 in 171 patients who had undergone colorectal adenocarcinoma surgery. We found strong correlations between Wnt2 expression and progression‐related variables such as tumor depth and lymph node metastasis.

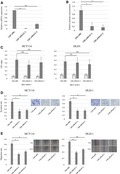

## 1 INTRODUCTION

Colorectal cancer (CRC) is one of the most common types of cancer globally. Treatments for patients with CRC have recently made steady progress, such as chemotherapy, surgery, and neoadjuvant/adjuvant therapy.^1^, ^2^, ^3^, ^4^ However, it remains the third most common cancer and the fourth most common cause of cancer‐related death, with 700 000 deaths per year globally, exceeded only by lung, liver, and stomach cancers.^5^ There is thus an urgent need to develop a novel treatment method for CRC.

Recent studies have shown that stromal cells in cancer tissues control and contribute to tumor progression.^6^ Stromal cells including fibroblasts, immune cells, endothelial cells, and inflammatory cells organize the tumor microenvironment to affect the proliferation, invasion, and migration of cancer cells.^7^, ^8^ Cancer‐associated fibroblasts (CAFs), a major component of the tumor stroma, play a particularly significant part in tumor progression through communication with cancer cells in many solid tumors.^9^ CAFs have been found to promote tumor progression by a variety of oncological functions.^10^ For example, Gaggioli et al demonstrated that CAFs are required to promote the invasion of squamous cell carcinoma cells.^11^ CAFs have also been shown to induce tumor growth and angiogenesis in invasive breast carcinomas.^12^ In addition, in pancreatic cancer, CAFs promote chemoresistance of cancer cells through signaling mediated by exosomes.^13^ A study by Itoh et al also revealed that CAFs control apoptosis to promote cancer dissemination in gastric cancer cells.^14^ Considering the above, CAFs can be a critical mediator in cancer progression. However their underlying mechanism and clinical significance in CRC is not fully understood. Against this background, the purpose of this study was to explore the mechanisms of CAFs involvement in CRC progression in order to improve the prognosis of patients with CRC.

Here, analysis of the gene expression profiles of primary cancer‐associated and normal fibroblasts isolated from surgical specimens revealed that Wnt2 plays a critical role in colorectal CAFs. Furthermore, we examined the effects of Wnt2 expression on clinicopathological factors in patients with CRC and conducted in vitro analyses, which revealed that Wnt2 enhances cancer cell invasion and migration. *Wnt2* is a member of *Wnt* family and is located on human chromosome 7q31. Wnt2 protein is secreted glycoproteins and the ligand of the Wnt signaling pathway. Although Wnt2 has been reported to be associated with the regulation of tumorigenesis and the progression of various tumors,^15^, ^16^, ^17^, ^18^ few studies have focused on the interaction between cancer cells and CAFs based on Wnt2 proteins.^19^, ^20^ Our findings suggest that colorectal CAFs secrete Wnt2 to promote cancer progression, and strategy targeting of Wnt2 derived from CAFs is a potential management strategy for colorectal cancer.

## 2 MATERIAL AND METHOD

### 2.1 Cell lines and cell culture

Human colon carcinoma cell lines (HCT116, DLD‐1, and HT‐29) and human colon normal fibroblast cell line (CCD‐18Co) were obtained from the American Type Culture Collection. HCT116 and DLD‐1 were cultured in RPMI 1640 Medium (Invitrogen) and HT‐29 was in McCoy's 5A (Invitrogen). CCD‐18Co was cultured in Eagle's minimal essential medium. These media were supplemented with 10% heat‐inactivated fetal bovine serum (FBS; Biowest) and 1% penicillin‐streptomycin (Invitrogen).

### 2.2 Isolation of colon fibroblasts

CAFs and normal fibroblasts (NFs) were prepared by the outgrowth method.^21^, ^22^ These fibroblasts were obtained from freshly resected specimens from patients with CRC. Small tissue blocks were minced with scissors and incubated in Dulbecco's modified Eagle medium (DMEM; Invitrogen) containing 10% FBS and 1% penicillin and streptomycin at 37°C in an atmosphere containing 5% CO_2_. CAFs were established from the tumor tissue, and NFs were from non‐tumor tissues at least 5 cm away from the tumor margin. These fibroblasts were used for experiments between passages 2 and 5. In vitro experiments were performed using CAFs from patient No. 5 (see Table 1), which were immortalized by transfecting with lentivirus encoding human telomerase reverse transcriptase. It is because primary CAFs may decrease proliferative potency and lose the unique characteristics of CAFs through passages. This study was approved by the Tohoku University ethics committee, and informed consent was obtained from the patients prior to the study.

**Table 1:** Characteristics of patients whose colorectal tissues were examined for isolating primary fibroblasts

No.	Age	Gender	Location of cancer	Histological differentiation	TNM classification
1	73	Male	Rectal cancer	Well differentiated	T3N1bM1a StageIVA
2	68	Male	Rectal cancer	Moderate differentiated	T3N1bM1a StageIVA
3	58	Female	Rectal cancer	Moderate differentiated	T3N2bM0 StageIIIC
4	66	Male	Rectal cancer	Moderate differentiated	T4aN0M0 StageIIB
5	61	Male	Rectal cancer	Moderate differentiated	T4aN2aM1a StageIVA

### 2.3 Quantitative real‐time PCR (qRT‐PCR)

RNA was extracted using the RNeasy Mini Kit (Qiagen) and cDNA was synthesized with PrimeScript RT‐PCR Kit (Takara Bio). qRT‐PCR was carried out using SYBR Premix Ex Taq II, ROX Plus (Takara Bio) on an ABI StepOne Plus (Life Technologies), in accordance with the manufacturers’ protocols. Relative quantification of mRNA within the samples was performed using the $2^{-\Delta \Delta C_{t}}$ method, and the results were normalized relative to the glyceraldehyde 3‐phosphate dehydrogenase (GAPDH) mRNA level. Triplicate samples were used in each experiment. The primer sequences used in this study were as follows: *CDH1* (E‐cadherin): forward 5′‐TTTGTACAGATGGGGTCTTGC‐3′ and reverse 5′‐CAAGCCCACTTTTCATAGTTCC‐3′, *ATCA2* (αSMA; alpha‐smooth muscle actin): forward 5′‐TAGAACACGGCATCATCA‐3′ and reverse 5′‐CCAGAGTCCAGCACAATA‐3′, *VIM* (vimentin): forward 5′‐TGGCACGTCTTGACCTTGAA‐3′ and reverse 5′‐GGTCATCGTGATGCTGAGAA‐3′, *FAP* (fibroblast activation protein alpha): forward 5′‐TGGGAATATTACGCGTCTGTCTAC‐3′ and reverse 5′‐GATAAGCCGTGGTTCTGGTCA‐3′, *WNT2* (wingless‐type MMTV integration site family, member 2): forward 5′‐CCAGCCTTTTGGCAGGGTC‐3′ and reverse 5′‐GCATGTCCTGAGAGTCCATG‐3′, and *GAPDH*: forward 5′‐GCACCGTCAAGGCTGAGAAC‐3′ and reverse 5′‐TGGTGAAGACGCCAGTGGA‐3′.

### 2.4 RNA sequencing (RNA‐seq)

RNA‐seq was conducted as described previously.^23^ An RNA‐seq library was prepared from total RNA using a TruSeq RNA Sample Prep Kit v2 (Illumina). Sequencing was performed using a HiSeq 2500 instrument (Illumina). The quality of the reads was evaluated with FastQC (http://www.bioinformatics.babraham.ac.uk/projects/fastqc). For gene expression analysis, single‐end reads were mapped to the human genome (Ensembl, release 74) with TopHat (ver. 2.1.0). Cufflinks (ver. 2.2.1) was used to estimate the gene expression level on the basis of fragments per kilobase of transcript per million mapped reads.

### 2.5 Analysis of gene expression profiling

To define differentially expressed genes, the combined thresholds of *P* < .05 and fold change >2 or <0.5 were used. We compared gene expression profiles of CAFs and NFs using Gene Ontology (GO) analysis and pathway analysis. We used the software Gene Set Enrichment Analysis (GSEA) v3.0 provided by the Broad Institute (http://www.broadinstitute.org/gsea/) for these analyses.^24^, ^25^ The gene set databases c5.all.v6.2.symbols.gmt and c2.cp.kegg.v6.2.symbols.gmt were applied for the GO analysis and KEGG pathway analysis, respectively.

### 2.6 Tissue specimens

This study included 171 patients who underwent surgery for colorectal adenocarcinoma from 2009 to 2012 at Tohoku University Hospital. None of these patients underwent preoperative chemotherapy and/or radiotherapy. Patients who had cancer associated with inflammatory bowel disease or familial adenomatous polyposis, anal canal carcinomas, double cancers, and remnant carcinoma after endoscopic resection were not enrolled in this study. Informed consent was obtained from all of the patients, and the Tohoku University ethics committee approved the research protocols for this study.

### 2.7 Immunohistochemistry (IHC)

Formalin‐fixed, paraffin‐embedded sections were cut from the tissues at a thickness of 3 µm. Paraffin sections were deparaffinized in xylene and rehydrated with graded ethanol to distilled water. Antigen retrieval was not performed. The nonspecific binding sites were blocked with 1% bovine serum albumin (Invitrogen) in phosphate‐buffered saline (PBS) for 30 minutes. Then, samples were incubated with rabbit anti‐Wnt2 polyclonal antibody (OriGene; dilution 1:300) as the primary antibodies, overnight at 4°C. After blocking the endogenous peroxidase activity by methanol containing 0.3% hydrogen peroxide (H_2_O_2_), the sections were sequentially incubated with EnVision+ System‐HRP (Dako) and the immune complexes were visualized with 3,3′‐diaminobenzidine (DAB: Dojindo, Kumamoto, Japan) solution [1 mmol/L DAB, 50 mmol/L Tris‐HCl buffer (pH 7.6), and 0.006% H_2_O_2_]. Finally, the sections were lightly counterstained with hematoxylin. Negative controls were established by omitting the primary antibody, and no detectable staining was evident.

For evaluating Wnt2 expression in CAFs, staining in fibromatous tumor stroma was graded as negative to weak (grade 0), moderate (grade 1), or strong (grade 2), considering the intensity of staining and the proportion of the positive area. Tumor stroma contains various types of cells, such as vascular endothelial cells, lymphocytes, and macrophages, in addition to fibroblasts. Therefore, it was a challenge to evaluate and calculate the exact proportion of positive fibroblasts. The intensity of staining in fibroblasts tended to increase in proportion to the spread of the cells. In addition, considering the ratio, the scoring was based on the intensity of immunoreactivity. Samples were classified into two groups for statistical analysis according to the staining score: grade 0 was defined as the low‐expression group, and grades 1 and 2 were defined as the high‐expression group.

### 2.8 RNA interference

Two siRNAs (Invitrogen) for downregulating *WNT2* were transfected into immortalized CAFs using Lipofectamine RNAiMAX transfection reagent (Invitrogen), in accordance with the manufacturer's instructions. The target sequences of siRNA were as follows: siWnt2‐1 (HSS111349), 5′‐UCACUGUGGCUAACGAGAGGUUUAA‐3′ (sense) and 5′‐UUAAACCUCUCGUUAGCCACAGUGA‐3′ (anti‐sense); and siWnt2‐2 (HSS187691), 5′‐AAGUAGUCGGGAAUCUGCCUUUGUU‐3′ (sense) and 5′‐AACAAAGGCAGAUUCCCGACUACUU‐3′ (anti‐sense). A non‐targeting siRNA was used as a negative control (siNC).

### 2.9 Preparation of conditioned medium

Immortalized CAFs derived from patient No. 5 were incubated to confluence in DMEM containing 10% FBS in a six‐well plate for 72 hours after the transfection of siRNA. Immortalized CAFs were washed with PBS and incubated for an additional 72 hours in 1 mL of DMEM without FBS. The culture medium was collected and centrifuged at 3000 *g* for 5 minutes to remove cell debris. The supernatant was stored at −30°C and used as a conditioned medium (CM). We prepared CM derived from immortalized CAFs transfected with siWnt2‐1 (CM‐siWnt2‐1), siWnt2‐2 (CM‐siWnt2‐2), and siNc (CM‐siNC).

### 2.10 Enzyme‐linked immunosorbent assay (ELISA)

Protein levels of Wnt2 in CM were measured using an ELISA kit (SEL820HU; Cloud‐Clone), in accordance with the manufacturer's protocol.

### 2.11 Cell proliferation assay

Cell proliferation activity was assessed by MTS assay using CellTiter 96® AQueous One Solution Reagent (Promega). DLD‐1 (4000 cells) or HCT116 (6000 cells) was seeded in 96‐well plates and incubated in RPMI 1640 with 10% FBS for 24 hours. Cells were washed with PBS and incubated in CM supplemented with 2% FBS for an additional 96 hours. Before and after incubation in CM, 50 µL of MTS solution was added into each well and incubated for 1 hours; absorbance was measured using a Multiskan FC plate reader (Thermo Fisher Scientific) at 490 nm.

### 2.12 Invasion assay

For the invasion assay, we used a 24‐well Transwell chamber containing a polycarbonate filter with 8‐μm pores (Corning Inc). Cancer cells were seeded to inserts with or without Matrigel coating (BD Biosciences) in 0.5 mL of serum‐free DMEM. The lower well was filled with 0.5 mL of CM supplemented with 10% FBS. After 48 hours, cells invading to the bottom surface of the membrane were fixed and stained. These cells were counted in five randomly selected areas under a 200× microscope. Data are expressed as the percent invasion through the membrane with Matrigel coating relative to the migration through the control membrane without Matrigel coating, in accordance with the manufacturer's protocol.

### 2.13 Wound healing assay

Migration of cancer cells was measured by wound healing assay. HCT116 and DLD‐1 were seeded in 24‐well plates. Cells were incubated to confluence for 24 hours, and the cell monolayer was scratched with a 200‐µL pipette tip to create a wound. After the cells had been washed with PBS, CM supplemented with 2% FBS was added to the wells and cells were incubated for 24 hours. The cell migration was measured with Axio Vs 40 v4.8.2.0 (Carl Zeiss, Oberkochen, Germany) and is expressed as the rate of wound healing (=1 − blank area at 24 h/blank area at 0 h) as per a previous report.^26^

### 2.14 Statistical analysis

Data are expressed as mean ± SEM. The significance of differences was analyzed using Student's *t* test in the in vitro experiments. All experiments were repeated three times. In the immunohistochemical studies, the correlation between the expression level of the protein and clinicopathological factors was analyzed using the *χ* ^2^ test or Student's *t* test. Survival curves were constructed using the Kaplan‐Meier method and evaluated using the log‐rank test. Values of *P* < .05 were considered statistically significant. All analyses were performed using JMP 13 software (SAS Institute Inc).

## 3 RESULT

### 3.1 Isolation of CAFs and NFs from human colon tissues

**Figure 1:**
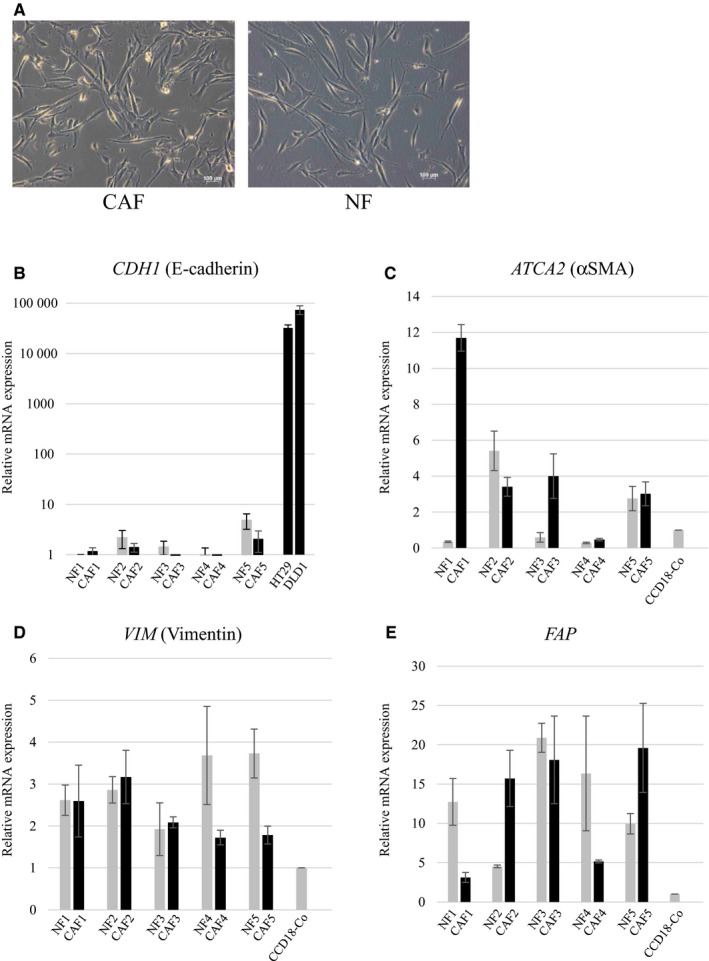
Fibroblasts isolated from human colorectal cancer tissues. Isolated cells showed a spindle‐like shape, which makes no morphological distinction between CAFs and NFs (A). They were negative for the epithelial marker E‐cadherin (B). The expression of αSMA, vimentin, and FAP in these cells did not show a specific pattern between CAFs and NFs (C‐E)

The pairs of CAFs and NFs were isolated from the specimens obtained from five patients with CRC (Table 1). These cells showed a spindle‐like shape, which is typical of fibroblasts (Figure 1A). They had an extremely low level of E‐cadherin mRNA expression, as an epithelial marker, in qRT‐PCR (Figure 1B). The results indicated that fibroblast cells were appropriately isolated from tumor tissue and normal mucosa. We also examined the expression of αSMA, vimentin, and FAP in these cells as fibroblast markers. However, the expression of these markers did not show a specific pattern between CAFs and NFs (Figure 1C‐E).

### 3.2 RNA‐seq analysis

**Figure 2:**
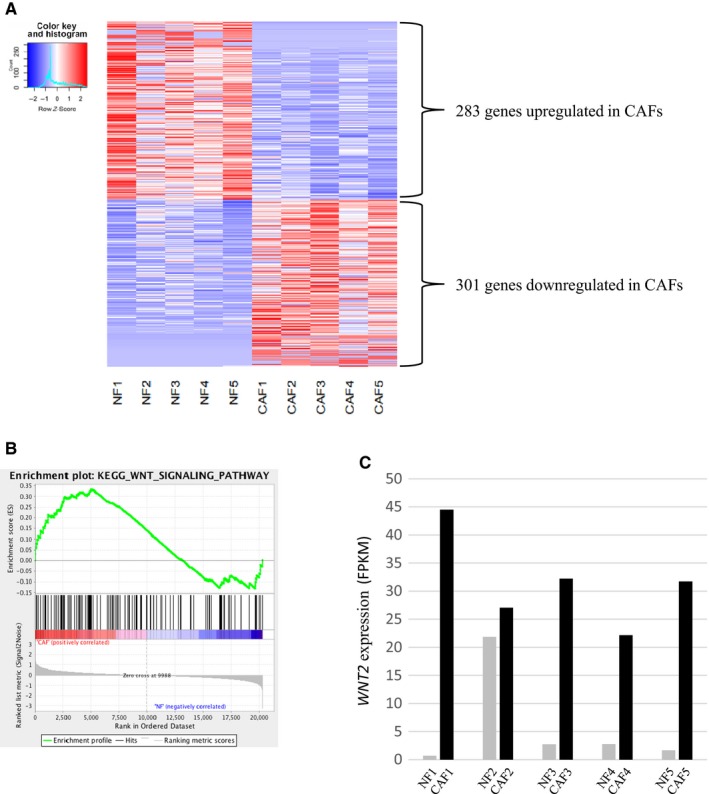
Analysis of gene expression profiling. The heat map demonstrated differentially expressed genes identified with RNA sequencing, including 283 upregulated genes and 301 downregulated genes in CAFs (A). Pathway analysis using Gene Set Enrichment Analysis revealed that the gene sets related to the Wnt signaling pathway were highly enriched in CAFs (B). CAFs had much higher *WNT2* expression than NFs among the primary fibroblasts from each of the five cases (C). FPKM: fragments per kilobase of transcript per million mapped reads

Gene expression profiles were obtained from five pairs of CAFs and NFs isolated by primary culture. The analysis of gene expression profiles identified 584 genes differentially expressed between CAFs and NFs, including 283 upregulated genes and 301 downregulated genes in CAFs (Figure 2A). GO analysis and pathway analysis revealed that plenty of gene sets related to the Wnt signaling pathway were highly enriched in CAFs, as well as the TGFβ signal pathway, which is considered as a trigger of differentiation to CAFs from NFs (Figure 2B; Tables 2 and S1).^27^ To explore the genes playing important roles in Wnt signaling in CAFs, we analyzed the expression of each gene within the KEGG Wnt signaling pathway with GSEA. Among them, Wnt2 had the highest ranking in the gene list and the highest rank metric score regarding the differential expression between CAFs and NFs (Table 3). This means that Wnt2 was specifically expressed in CAFs compared with other genes within the Wnt signaling pathway. Indeed, CAFs exhibited much higher *WNT2* expression than NFs in primary fibroblasts from each of the five cases (Figure 2C). Therefore, it was assumed that Wnt2 served as a critical mediator for inducing CRC progression by CAFs.

**Table 2:** KEGG Pathway analysis

Name	SIZE	ES	NES	NOM *P*‐val	FDR q‐val
KEGG_PROTEASOME	41	0.56163	1.9084287	0	0.025224
KEGG_ARRHYTHMOGENIC_RIGHT_VENTRICULAR_CARDIOMYOPATHY_ARVC	70	0.488159	1.8073922	0	0.042101
KEGG_PATHOGENIC_ESCHERICHIA_COLI_INFECTION	53	0.484691	1.7336141	0	0.062389
KEGG_REGULATION_OF_ACTIN_CYTOSKELETON	199	0.377994	1.669645	0	0.09594
KEGG_FOCAL_ADHESION	193	0.375368	1.6675874	.002551	0.078578
KEGG_HEDGEHOG_SIGNALING_PATHWAY	51	0.471096	1.6389468	.012245	0.085571
KEGG_ECM_RECEPTOR_INTERACTION	80	0.428931	1.6388332	.008929	0.073641
KEGG_VIRAL_MYOCARDITIS	64	0.431181	1.6267112	.002268	0.07074
KEGG_CELL_ADHESION_MOLECULES_CAMS	114	0.385292	1.6078509	0	0.072959
KEGG_SMALL_CELL_LUNG_CANCER	84	0.394938	1.5541589	.011261	0.103223
KEGG_BASAL_CELL_CARCINOMA	50	0.436296	1.5480132	.010246	0.098083
KEGG_LEUKOCYTE_TRANSENDOTHELIAL_MIGRATION	106	0.37584	1.5435936	0	0.093158
KEGG_THYROID_CANCER	29	0.480652	1.5274546	.023504	0.097032
KEGG_P53_SIGNALING_PATHWAY	66	0.407492	1.5242089	.016878	0.092584
KEGG_HYPERTROPHIC_CARDIOMYOPATHY_HCM	75	0.392986	1.5123453	.002193	0.095073
KEGG_RENAL_CELL_CARCINOMA	69	0.3904	1.4776138	.014925	0.118615
KEGG_TIGHT_JUNCTION	123	0.352359	1.4742628	.006772	0.11432
KEGG_WNT_SIGNALING_PATHWAY	140	0.333361	1.4278333	.008969	0.154247
KEGG_TGF_BETA_SIGNALING_PATHWAY	83	0.353121	1.4017543	.031674	0.176525
KEGG_ADHERENS_JUNCTION	73	0.352914	1.3431549	.059211	0.248549
KEGG_GLYCOSAMINOGLYCAN_BIOSYNTHESIS_HEPARAN_SULFATE	24	0.440585	1.3352597	.112798	0.249587
KEGG_MELANOGENESIS	93	0.332118	1.3331608	.051836	0.241666

SIZE: Number of genes in the gene set after filtering out those genes not in the expression dataset.

ES: Enrichment score for the gene set; that is, the degree to which this gene set is overrepresented at the top or bottom of the ranked list of genes in the expression dataset.

NES: Normalized enrichment score; that is, the enrichment score for the gene set after it has been normalized across analyzed gene sets.

NOM *P*‐val: Nominal *P* value; that is, the statistical significance of the enrichment score. The nominal *P* value is not adjusted for gene set size or multiple hypothesis testing; therefore, it is of limited use in comparing gene sets.

FDR q‐value: False discovery rate; that is, the estimated probability that the normalized enrichment score represents a false positive finding.

**Table 3:** Top 20 genes in KEGG Wnt signaling pathway

Name	Rank in gene list	Rank metric score	Running ES
WNT2	24	1.482293	0.03164
SFRP2	32	1.371907	0.061677
WNT5A	138	1.013468	0.078918
PRICKLE1	183	0.969715	0.098214
VANGL2	256	0.896152	0.114493
LEF1	448	0.784525	0.1224
CCND1	452	0.781046	0.13955
PLCB4	717	0.684703	0.141628
LRP6	720	0.683883	0.156675
FZD7	852	0.64638	0.164498
PRKACG	926	0.625185	0.174726
WNT4	969	0.616328	0.186294
WNT11	1159	0.572646	0.189608
PORCN	1171	0.570418	0.201696
SFRP4	1177	0.567706	0.214022
PPP2R5B	1460	0.509164	0.21132
FZD10	1696	0.471635	0.210117
FZD8	1718	0.468426	0.21945
CCND3	1841	0.450994	0.223392
FZD4	1949	0.444956	0.227942

Rank in Gene List is the position of the gene in the ranked list of all genes included in our gene expression profile.

Rank Metric Score refers to the signal to noise ratio for each gene used to position the gene in the ranked list.

Running ES is the enrichment score at this point in the ranked list of genes.

### 3.3 Correlations between Wnt2 expression in cancer stroma and clinicopathological features

**Figure 3:**
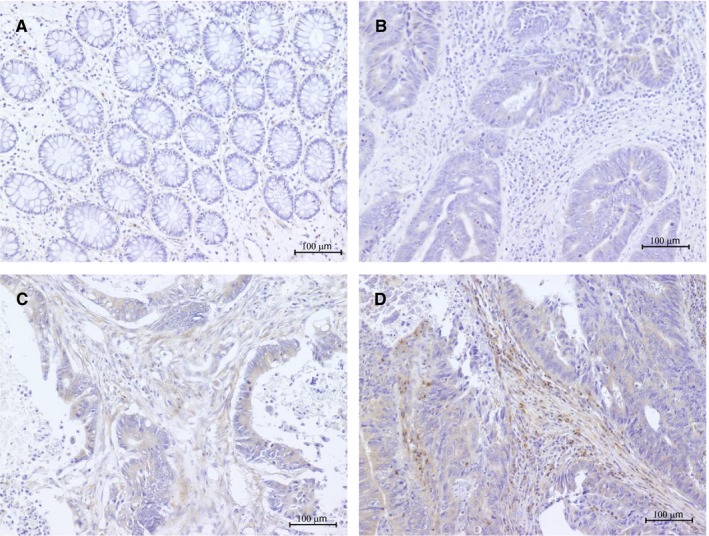
Wnt2 expression in colon tissue by immunohistochemistry. The images show representative immunohistochemical staining in normal colon tissue (A), cancer tissue with a score of 0 (B), cancer tissue with a score of 1 (C), and cancer tissue with a score of 2 (D). Immunoreactivity was found in cancer stroma, particularly in the fibromatous area, rather than cancer calls, although staining for Wnt2 were rarely observed in both epithelial and stromal cells in normal tissues

**Figure 4:**
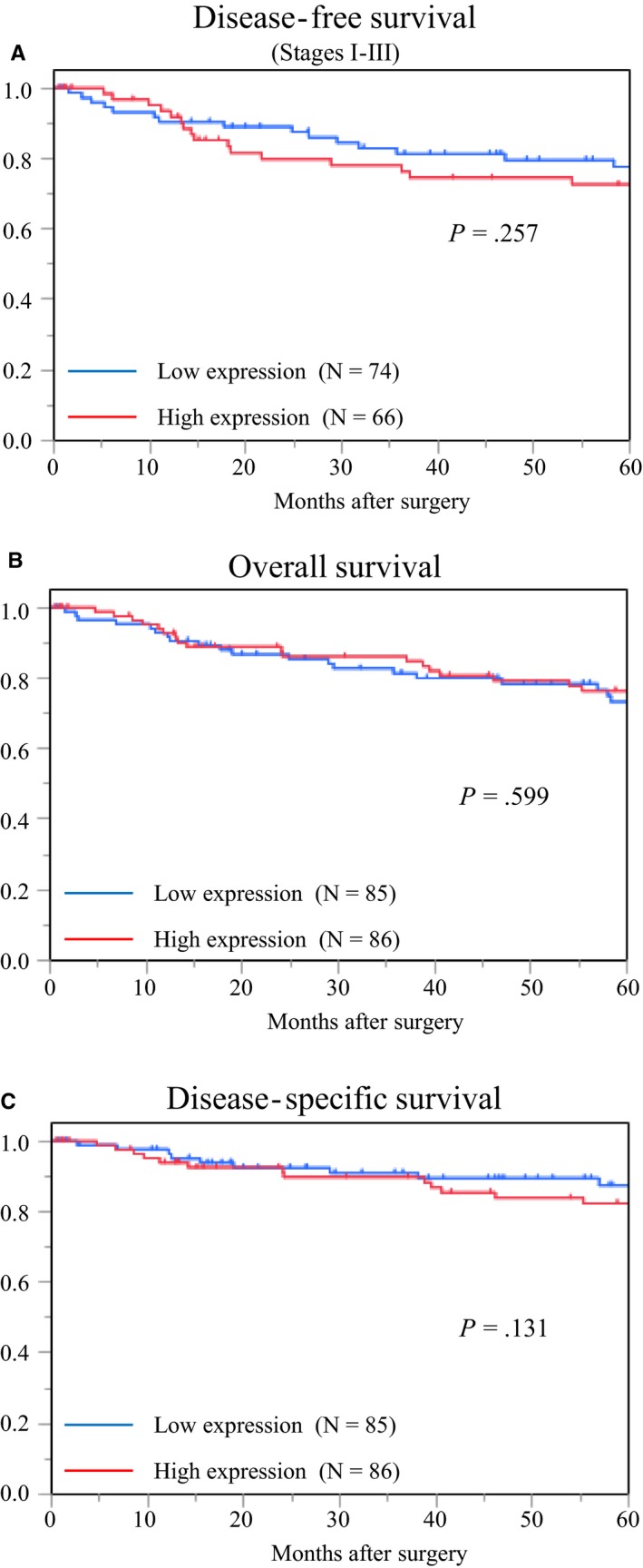
Kaplan‐Meier analysis of disease‐free survival (A), overall survival (B), and disease‐specific survival (C) of patients with colorectal cancer according to the expression of Wnt2 protein. There was no significant correlation between the high‐expression group and the low‐expression group in these variables

Immunohistochemical analysis was performed to examine the expression of Wnt2 in CRC tissue samples. In normal mucosa, cells stained for Wnt2 were rarely observed in both epithelial and stromal tissues (Figure 3A). Positive staining was often observed in cancer stroma, particularly in the fibromatous area. Strong staining was mainly detected in fibromatous cancer stroma in the deep regions. However, immunoreactivity for Wnt2 was native or weak in cancer cells. In the cases with a score of 0, few positive fibroblasts were found (Figure 3B). The staining of fibroblasts in tumor stroma was clearly observed in the patients with a score of 1 (Figure 3C); although in the cases that exhibited stronger intensity and wider distribution of immunoreactivity in fibromatous cancer stroma than others, the patients scored 2 (Figure 3D). To assess the Wnt2 expression level in CAFs, the staining in fibromatous tumor stroma was evaluated, excluding cancer cells; 171 samples were classified into the low‐expression group (85/171, 49.7%) or high‐expression group (86/171, 50.3%). Wnt2 expression in CAFs was significantly correlated with the depth of tumor invasion (*P* < .001), lymph node metastasis (*P* = .044), TNM stage (*P* = .010), venous invasion (*P* < .001), and recurrence (*P* = .013) (Table 4). Kaplan‐Meier survival analysis indicated that there was no significant correlation between Wnt2 expression in CAFs and disease‐free survival (Figure 4A), overall survival (Figure 4B), or disease‐specific survival (Figure 4C).

**Table 4:** Correlations between Wnt2 expression and clinicopathological factors in patients with colorectal cancer

Clinicopathological factors	Low expression (N = 85)	High expression (N = 86)	*P* value
Age (y)^a^	69 (35‐92)	69 (34‐89)	.461
Gender			.826
Male	49	51	
Female	36	35	
Location of tumor			.953
Right side	32	32	
Left side	53	54	
Size (mm)^a^	31 (9‐130)	39.5 (7‐125)	.110
Histologic differentiation			.199
Well or Moderate differentiated	82	79	
Others	3	7	
Depth of tumor			**<.001**
T1 + T2	42	14	
T3 + T4	43	72	
Lymph node metastasis			**.044**
N0	61	49	
N1 + N2	24	37	
Distant metastasis			.080
M0	74	66	
M1	11	20	
TNM Stage			**.010**
I + II	57	41	
III + IV	28	45	
Lymphatic invasion			.303
No	31	25	
Yes	54	61	
Venous invasion			**<.001**
No	27	8	
Yes	58	78	
Recurrence (Stage I‐III)			**.013**
No	69	52	
Yes	5	14	

a The values were presented as median (range) and analyzed using *χ* ^2^ test. All other values represented the number of cases and analyzed using Student's *t* test. *P* < .05 was considered significant.

*P* < .05 was defined as significant, and listed in bold.

### 3.4 CAF‐secreted Wnt2 promotes CRC cell proliferation, migration, and invasion

**Figure 5:**
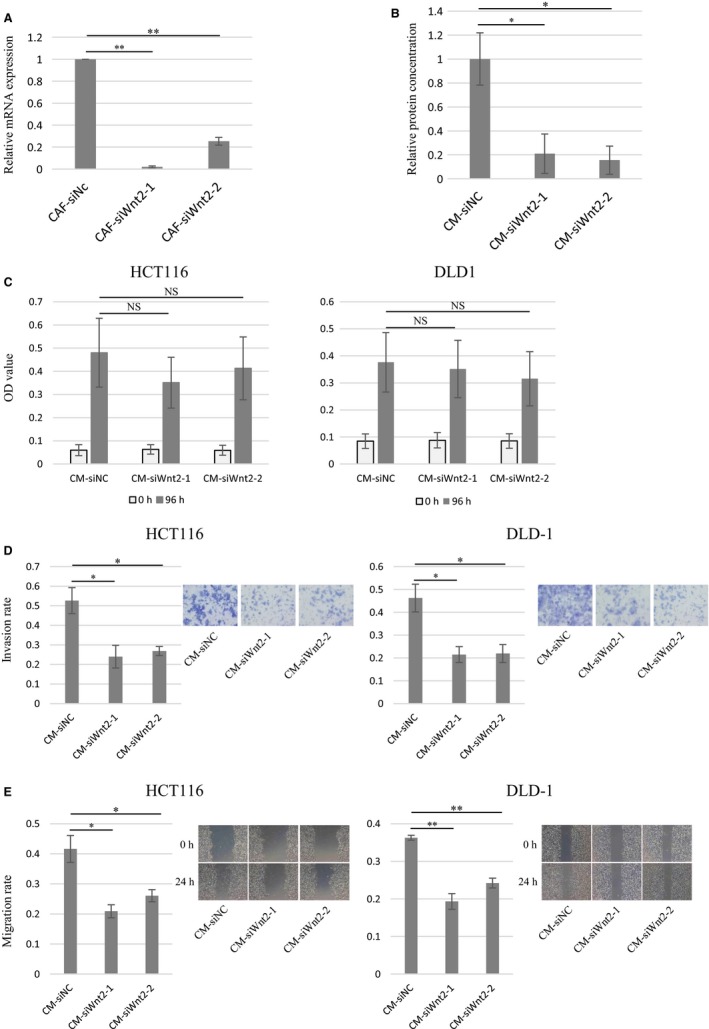
Wnt2 protein secreted by CAFs promotes the invasion and migration of colorectal cancer cells in vitro. The qRT‐PCR analysis confirmed the downregulation of the Wnt2 mRNA expression levels in immortalized CAFs transfected with siRNA targeting Wnt2 compared with that in those transfected with non‐targeting siRNA (A). ELISA showed that conditioned medium (CM) from immortalized CAFs transfected with siRNA targeting Wnt2 contained lesser Wnt2 protein than that with non‐targeting siRNA (B). Although there was no difference in cancer cell progression among the three types of CM (C), cancer cell invasion and migration significantly decreased in CM from CAFs transfected with siRNA targeting Wnt2 (D, E). CM‐siWnt2‐1: conditioned medium derived from CAFs transfected with siWnt2‐1, CM‐siWnt2‐2: conditioned medium derived from CAFs transfected with siWnt2‐2, CM‐siNC: conditioned medium derived from CAFs transfected with non‐targeting siRNA. *P < .05 and **P < .01 versus control (left bar)

The histopathological study suggested that Wnt2 protein was overexpressed in CAFs and involved in CRC progression. Thus, in vitro experiments were conducted to explore how Wnt2 induced tumor progression through the interaction between cancer cells and CAFs in CRC. First, siRNA targeting Wnt2 was transfected into immortalized CAFs. The qRT‐PCR analysis confirmed the downregulation of the Wnt2 mRNA expression levels in immortalized CAFs transfected with siWnt2‐1 and siWnt2‐2 compared with that in those transfected with siNC at 48 hours after transfection (Figure 5A). We conducted ELISA to measure the amount of Wnt2 protein in CM derived from immortalized CAFs transfected with siRNA. The result showed that CM‐siWnt2‐1 and CM‐siWnt2‐2 contained less Wnt2 protein than CM‐siNC (Figure 5B).

Subsequently, to evaluate the biological functions of Wnt2, in vitro experiments were performed using CM. In the MTS assay, there was no difference in cancer progression for 96 hours of incubation among the three types of CM (Figure 5C). However, cancer cell (DLD‐1 and HCT116) invasion was significantly decreased in CM‐siWnt2‐1 and CM‐siWnt2‐2 compared with that in CM‐siNC (Figure 5D). Cancer cells similarly exhibited decreased migration in CM‐siWnt2‐1 and CM‐siWnt2‐2 in the wound healing assay (Figure 5E). These results showed that CAFs secreted Wnt2 protein, which promoted CRC cell invasion and migration.

## 4 DISCUSSION

Recent studies have reported that a variety of signals by cytokines, chemokines, or growth factors derived from CAFs affect the progression of solid cancers.^28^, ^29^, ^30^ In this study, to elucidate the mechanisms by which CAFs regulate CRC progression, we focused on Wnt2, which was found to be highly expressed in CAFs compared with the level in NFs, by RNA‐seq analysis.

First, we performed a primary culture of CAFs and NFs from resected colorectal specimens. Their spindle shape and extremely low expression of epithelial markers showed that the cells that we established in primary culture were not contaminated with epithelial cells. The expression of fibroblast markers was measured by qRT‐PCR in CAFs, NFs, and CCD18‐Co, although they did not distinguish between these cell types. Nonetheless, these cells exhibited different patterns of αSMA, vimentin, and FAP expression. These findings match those reported in a previous study.^31^ In fact, various studies have recently reported the heterogeneity of CAFs.^32^ Our findings also suggested that the expression of αSMA, vimentin, and FAP does not necessarily represent activated fibroblasts, and thus, in this study, CAFs, NFs, and CCD18‐Co could not be identified on the basis of the expression of these genes. Dykes et al proposed that, although several markers have been utilized to target CAFs, there is a lack of markers with specificity for CAF, which makes identifying CAFs challenging.^33^ However, more than 500 genes were found to be differentially expressed between CAFs and NFs in the RNA‐seq analysis, despite them being obtained from the same specimens. Furthermore, GSEA revealed that gene sets related to the TGFβ signaling pathway, EMT, or interleukin, which were previously reported to be associated with CAFs, were upregulated in CAFs.^34^, ^35^, ^36^ These findings suggest that we had correctly isolated CAFs and NFs.

GO analysis and KEGG pathway analysis detected various gene sets related to the Wnt signaling pathway that were highly expressed in CAFs. It indicated that the Wnt signaling pathway has a key role in CAFs of CRC and is involved in the interactions between cancer cells and CAFs. Among the genes included within the KEGG Wnt signaling pathway, Wnt2 had the highest rank in the gene list and the highest rank metric score. This meant that Wnt2 was specifically expressed in CAFs compared with the other genes in the Wnt signaling pathway, which suggested that Wnt2 has a greater impact on the Wnt signaling pathway in CAFs. Therefore, Wnt2 was picked out as a candidate gene for involvement in the regulation of CRC progression by CAFs.

To clarify how Wnt2 in CAFs contributes to CRC progression, we performed histopathological studies and experiments in vitro. In the immunohistochemical examination, positive staining was often observed in fibromatous cancer stroma. We assessed staining in this area to determine the expression of Wnt2 in CAFs. In the present study, Wnt2 expression in CAFs was significantly associated with factors leading to cancer progression, such as depth of tumor invasion, lymph node metastasis, TNM stage, vascular invasion, and recurrence. The primary CAFs, which were used in the present study, were isolated only from rectum, and our RNA‐seq results might reflect the features of left‐sided colorectal cancer. However, positive staining for Wnt2 was equally observed in right and left side tumor. Consequently, molecular profiles, at least of Wnt2, were considered independent of the tumor location.

Next, in vitro experiments were performed using CM from immortalized CAFs transfected with siWnt2. There were lower levels of Wnt2 protein in CM‐siWnt2‐1 and CM‐siWnt2‐2 with a decreasing mRNA level of Wnt2 in immortalized CAFs. Cancer cell invasion and migration were significantly decreased in CM‐siWnt2‐1 and CM‐siWnt2‐2 compared with those in CM‐siNC. These findings demonstrated that CAFs secreted Wnt2 protein in a paracrine fashion and promoted cancer cell invasion and migration in CRC. The results from the in vitro study were consistent with the results from immunohistochemical examination.

Various previous studies focused on the process or pathway that regulates tumor progression involving CAFs. In particular, some studies reported the relationship between CAFs and Wnt signaling.^31^, ^37^, ^38^ The Wnt proteins are a large family of secretory glycoproteins that regulate a variety of biological and developmental processes through multiple signals, including canonical Wnt/β‐catenin, Wnt/Ca^2+^, and the planar cell polarity pathway.^39^, ^40^ The Wnt signaling pathway has been shown to be involved in cancer progression.^17^, ^41^, ^42^ However, many of the mechanisms of cancer progression that CAFs control through the Wnt pathway remain poorly understood.

Among various Wnt proteins, Wnt2 protein is rich in cysteine residues and is approximately 40 kDa in size, containing about 360 amino acids^19^; it activates the canonical Wnt/β‐catenin pathway. Previous studies showed that Wnt2 is upregulated in some cancers and leads to cancer progression. For instance, Wnt2 promotes the progression of non‐small cell lung cancer through activating the Wnt/β‐catenin pathway.^16^ Wnt2 also activates the Wnt/β‐catenin pathway and induces cancer metastasis in pancreatic cancer and cervical cancer.^17^, ^18^ Moreover, CAFs have recently attracted attention as a source of Wnt2 secretion to promote tumor development. A study by Fu et al showed that Wnt2 was highly expressed in fibroblasts of esophageal cancer stroma. It also demonstrated that CAFs secreted Wnt2 protein in a paracrine fashion, which activated the Wnt/β‐catenin signaling pathway in cancer cells to promote tumor progression in esophageal cancer.^20^ In addition, Xu et al^19^ showed that Wnt2 protein from pancreatic stellate cells enhanced the migration and invasion of pancreatic cancer cells. In CRC tissue, CAFs were also shown to secrete growth factors that stimulate Wnt signaling activity in cancer cells.^43^ Kramer et al previously presented that Wnt2 expression of colorectal CAFs affected tumor invasion and metastasis from findings of in vitro and in vivo studies.^44^ In fact, they demonstrated that Wnt2 activated the autocrine canonical Wnt signaling pathway in fibroblast, which was associated with a pro‐migratory and pro‐invasive phenotype. Conversely, we focused on Wnt2 protein in culture media of CAFs enhancing the migration and invasion of cancer cells in CRC. Our study clearly revealed that CAFs release Wnt2 protein to cancer cells to promote CRC progression in a paracrine fashion. Moreover, we demonstrated the correlation between Wnt2 expression in colorectal CAFs and clinicopathological features using surgical human specimens.

In conclusion, the present study showed that Wnt2 expression in CAFs was significantly related to factors leading to tumor progression, such as depth of tumor invasion and lymph node metastasis, as revealed by immunochemical examination. We also demonstrated that Wnt2 protein derived from CAFs induced cancer cell migration and invasion in CRC. These findings suggest that Wnt2 secreted by CAFs is a critical mediator in CRC progression and is a potential therapeutic target for CRC.

## CONFLICT OF INTEREST

The authors have no conflict of interest.
