# Supplementary material for: Cancer‐associated fibroblasts secrete Wnt2 to promote cancer progression in colorectal cancer
Source: Cancer Med. 2019 Aug 29;8(14):6370–82. doi: 10.1002/cam4.2523 (PMC6797671; doi:10.1002/cam4.2523)
Supplement: Supplementary file 1 [file CAM4-8-6370-s001.docx]

Table S1. Gene Ontology analysis

| NAME | SIZE | ES | NES | NOM p-val | FDR q-val |
| --- | --- | --- | --- | --- | --- |
| GO_ANTIGEN_PROCESSING_AND_PRESENTATION_OF_PEPTIDE_ANTIGEN_VIA_MHC_CLASS_I | 86 | 0.545803 | 2.15718 | 0 | 0.014887 |
| GO_PROTEIN_BINDING_INVOLVED_IN_CELL_ADHESION | 16 | 0.745559 | 2.0344334 | 0 | 0.086737 |
| GO_VENTRICULAR_SEPTUM_DEVELOPMENT | 52 | 0.569577 | 2.0335505 | 0 | 0.057824 |
| GO_ANTIGEN_PROCESSING_AND_PRESENTATION_OF_EXOGENOUS_PEPTIDE_ANTIGEN_VIA_MHC_CLASS_I | 62 | 0.540713 | 2.0157928 | 0 | 0.058679 |
| GO_FILAMENTOUS_ACTIN | 19 | 0.697504 | 2.0043848 | 0 | 0.055832 |
| GO_ACTIN_FILAMENT_BUNDLE | 51 | 0.548944 | 1.968054 | 0 | 0.074033 |
| GO_EPITHELIAL_TO_MESENCHYMAL_TRANSITION | 54 | 0.544266 | 1.960258 | 0 | 0.071611 |
| GO_CELL_CELL_ADHERENS_JUNCTION | 54 | 0.528937 | 1.9555941 | 0 | 0.067512 |
| GO_EPHRIN_RECEPTOR_SIGNALING_PATHWAY | 83 | 0.499425 | 1.9550059 | 0 | 0.060263 |
| GO_POSITIVE_REGULATION_OF_INTERLEUKIN_8_PRODUCTION | 36 | 0.585652 | 1.9543945 | 0 | 0.054348 |
| GO_INTERMEDIATE_FILAMENT | 90 | 0.484832 | 1.9502816 | 0 | 0.053032 |
| GO_CARDIAC_SEPTUM_DEVELOPMENT | 82 | 0.490728 | 1.9372169 | 0 | 0.058871 |
| GO_ACTOMYOSIN | 55 | 0.52788 | 1.9302976 | 0 | 0.059676 |
| GO_NON_CANONICAL_WNT_SIGNALING_PATHWAY | 131 | 0.457565 | 1.9038103 | 0 | 0.077802 |
| GO_REGULATION_OF_T_CELL_CYTOKINE_PRODUCTION | 19 | 0.668966 | 1.9000177 | 0 | 0.075796 |
| GO_FACE_DEVELOPMENT | 48 | 0.534302 | 1.897239 | 0 | 0.073051 |
| GO_MESENCHYME_MORPHOGENESIS | 38 | 0.564062 | 1.887268 | 0.002336 | 0.077055 |
| GO_MICROFILAMENT_MOTOR_ACTIVITY | 18 | 0.662433 | 1.8833001 | 0.002155 | 0.076507 |
| GO_NEGATIVE_REGULATION_OF_CARTILAGE_DEVELOPMENT | 26 | 0.618115 | 1.8796662 | 0 | 0.075905 |
| GO_SARCOMERE_ORGANIZATION | 24 | 0.620077 | 1.8696818 | 0 | 0.081858 |
| GO_PITUITARY_GLAND_DEVELOPMENT | 34 | 0.570019 | 1.8672811 | 0.002041 | 0.080065 |
| GO_CARDIAC_VENTRICLE_DEVELOPMENT | 95 | 0.46373 | 1.8562036 | 0 | 0.087187 |
| GO_PROTEIN_COMPLEX_INVOLVED_IN_CELL_ADHESION | 30 | 0.582499 | 1.849809 | 0 | 0.089889 |
| GO_REGULATION_OF_DENDRITIC_SPINE_MORPHOGENESIS | 30 | 0.58596 | 1.8446598 | 0 | 0.091846 |
| GO_POSITIVE_REGULATION_OF_INTERFERON_BETA_PRODUCTION | 30 | 0.559588 | 1.8326908 | 0.00409 | 0.100976 |
| GO_HETEROTYPIC_CELL_CELL_ADHESION | 24 | 0.600404 | 1.8271377 | 0 | 0.103634 |
| GO_ANATOMICAL_STRUCTURE_MATURATION | 36 | 0.550482 | 1.8263764 | 0 | 0.100727 |
| GO_MUSCLE_TISSUE_DEVELOPMENT | 245 | 0.397337 | 1.8176289 | 0 | 0.108441 |
| GO_MYOSIN_FILAMENT | 18 | 0.641407 | 1.8161159 | 0.002033 | 0.106313 |
| GO_REGULATION_OF_PATHWAY_RESTRICTED_SMAD_PROTEIN_PHOSPHORYLATION | 54 | 0.504227 | 1.8145523 | 0 | 0.105092 |
| GO_HOMOTYPIC_CELL_CELL_ADHESION | 46 | 0.516182 | 1.8102616 | 0.002079 | 0.106945 |
| GO_POSITIVE_REGULATION_OF_PATHWAY_RESTRICTED_SMAD_PROTEIN_PHOSPHORYLATION | 43 | 0.516495 | 1.8100168 | 0 | 0.104133 |
| GO_CARTILAGE_DEVELOPMENT | 131 | 0.432992 | 1.8020021 | 0 | 0.110952 |
| GO_CHONDROCYTE_DEVELOPMENT | 21 | 0.611753 | 1.7941697 | 0.004184 | 0.117788 |
| GO_ACTIVIN_RECEPTOR_SIGNALING_PATHWAY | 19 | 0.624837 | 1.7918091 | 0.013304 | 0.117609 |
| GO_MESODERM_MORPHOGENESIS | 61 | 0.489067 | 1.7912271 | 0 | 0.115135 |
| GO_VENTRICULAR_SEPTUM_MORPHOGENESIS | 28 | 0.571881 | 1.786181 | 0.002128 | 0.118517 |
| GO_CARDIAC_CHAMBER_DEVELOPMENT | 130 | 0.427535 | 1.785346 | 0 | 0.116448 |
| GO_EPHRIN_RECEPTOR_ACTIVITY | 19 | 0.632929 | 1.7850809 | 0.014113 | 0.113697 |
| GO_POSITIVE_REGULATION_OF_CYTOKINE_PRODUCTION_INVOLVED_IN_IMMUNE_RESPONSE | 27 | 0.568382 | 1.7791744 | 0.002114 | 0.117936 |
| GO_CARDIAC_MUSCLE_TISSUE_DEVELOPMENT | 127 | 0.425501 | 1.776753 | 0 | 0.118342 |
| GO_POST_ANAL_TAIL_MORPHOGENESIS | 16 | 0.643469 | 1.7757496 | 0.010504 | 0.116663 |
| GO_ACTIN_FILAMENT_BUNDLE_ORGANIZATION | 46 | 0.509359 | 1.7721922 | 0 | 0.118925 |
| GO_HEART_VALVE_DEVELOPMENT | 31 | 0.553858 | 1.761892 | 0.002053 | 0.129672 |
| GO_PLATELET_AGGREGATION | 35 | 0.528753 | 1.7597655 | 0.002128 | 0.129851 |
| GO_POSITIVE_REGULATION_OF_WNT_SIGNALING_PATHWAY | 144 | 0.412327 | 1.7588599 | 0 | 0.128043 |
| GO_HETEROPHILIC_CELL_CELL_ADHESION_VIA_PLASMA_MEMBRANE_CELL_ADHESION_MOLECULES | 35 | 0.52808 | 1.7579763 | 0.006316 | 0.126581 |
| GO_CARDIAC_SEPTUM_MORPHOGENESIS | 47 | 0.498562 | 1.75638 | 0.002179 | 0.126392 |
| GO_ROUGH_ENDOPLASMIC_RETICULUM_MEMBRANE | 19 | 0.602876 | 1.746523 | 0.004193 | 0.13742 |
| GO_CELLULAR_DEFENSE_RESPONSE | 39 | 0.51947 | 1.7465051 | 0 | 0.134672 |
| GO_REGULATION_OF_CARTILAGE_DEVELOPMENT | 60 | 0.481241 | 1.7416515 | 0 | 0.139179 |
| GO_REGULATION_OF_INTERFERON_ALPHA_PRODUCTION | 19 | 0.605451 | 1.7369376 | 0.00202 | 0.142782 |
| GO_NEGATIVE_REGULATION_OF_EMBRYONIC_DEVELOPMENT | 26 | 0.561733 | 1.7341098 | 0.002309 | 0.144204 |
| GO_TOR_SIGNALING | 16 | 0.625871 | 1.7302483 | 0.010267 | 0.147298 |
| GO_FORMATION_OF_PRIMARY_GERM_LAYER | 100 | 0.425254 | 1.7294482 | 0 | 0.145784 |
| GO_METANEPHROS_MORPHOGENESIS | 24 | 0.570574 | 1.7288649 | 0.004073 | 0.144095 |
| GO_ENDOCARDIAL_CUSHION_DEVELOPMENT | 30 | 0.533233 | 1.7237678 | 0.004167 | 0.149091 |
| GO_REGULATION_OF_INTERLEUKIN_1_SECRETION | 25 | 0.563843 | 1.7229903 | 0.010395 | 0.147543 |
| GO_PANCREAS_DEVELOPMENT | 63 | 0.460266 | 1.7204531 | 0.002208 | 0.148639 |
| GO_ENDODERMAL_CELL_DIFFERENTIATION | 37 | 0.510682 | 1.720327 | 0 | 0.146408 |
| GO_POSITIVE_REGULATION_OF_NEURON_APOPTOTIC_PROCESS | 44 | 0.483545 | 1.714631 | 0.004246 | 0.152454 |
| GO_REGULATION_OF_INTERLEUKIN_8_PRODUCTION | 49 | 0.486809 | 1.7144336 | 0 | 0.150177 |
| GO_STRIATED_MUSCLE_CELL_DIFFERENTIATION | 151 | 0.400884 | 1.7120494 | 0 | 0.151012 |
| GO_CADHERIN_BINDING | 28 | 0.541731 | 1.7102815 | 0.004535 | 0.151146 |
| GO_HEAD_MORPHOGENESIS | 35 | 0.512086 | 1.7022257 | 0.002208 | 0.161046 |
| GO_CORTICAL_CYTOSKELETON | 76 | 0.439126 | 1.7006794 | 0.002141 | 0.160791 |
| GO_INTERMEDIATE_FILAMENT_CYTOSKELETON | 131 | 0.399438 | 1.6983275 | 0 | 0.162547 |
| GO_POTASSIUM_CHANNEL_REGULATOR_ACTIVITY | 38 | 0.504629 | 1.694759 | 0.004274 | 0.165318 |
| GO_REGULATION_OF_ACTION_POTENTIAL | 34 | 0.517644 | 1.6937377 | 0.002165 | 0.164196 |
| GO_DETECTION_OF_MECHANICAL_STIMULUS_INVOLVED_IN_SENSORY_PERCEPTION | 21 | 0.574386 | 1.6932248 | 0.019565 | 0.162566 |
| GO_ACTOMYOSIN_STRUCTURE_ORGANIZATION | 70 | 0.446517 | 1.6925387 | 0.006834 | 0.161221 |
| GO_NEUROTRANSMITTER_BINDING | 22 | 0.578726 | 1.6911302 | 0.006508 | 0.16093 |
| GO_POSITIVE_REGULATION_OF_TRANSMEMBRANE_RECEPTOR_PROTEIN_SERINE_THREONINE_KINASE_SIGNALING_PATHWAY | 93 | 0.422474 | 1.6865467 | 0 | 0.165656 |
| GO_KINESIN_COMPLEX | 53 | 0.470812 | 1.6858889 | 0.008715 | 0.164418 |
| GO_REGULATION_OF_INTERFERON_BETA_PRODUCTION | 42 | 0.491992 | 1.6843373 | 0.004425 | 0.164581 |
| GO_CARDIAC_CHAMBER_MORPHOGENESIS | 91 | 0.422545 | 1.6831028 | 0 | 0.164322 |
| GO_ACTIN_FILAMENT | 66 | 0.448209 | 1.6809397 | 0.002141 | 0.165417 |
| GO_DENDRITIC_SPINE_ORGANIZATION | 16 | 0.606029 | 1.6789889 | 0.01919 | 0.166348 |
| GO_PROTEIN_LOCALIZATION_TO_CENTROSOME | 15 | 0.622232 | 1.6776983 | 0.025157 | 0.166233 |
| GO_REGULATION_OF_ESTABLISHMENT_OF_PLANAR_POLARITY | 107 | 0.405963 | 1.6731577 | 0 | 0.170817 |
| GO_NEGATIVE_REGULATION_OF_CHONDROCYTE_DIFFERENTIATION | 20 | 0.582893 | 1.673111 | 0.008439 | 0.168779 |
| GO_PHOSPHATIDYLINOSITOL_3_KINASE_SIGNALING | 23 | 0.555629 | 1.6728497 | 0.011161 | 0.167097 |
| GO_CALCIUM_DEPENDENT_PHOSPHOLIPID_BINDING | 49 | 0.469266 | 1.6689235 | 0.002227 | 0.171609 |
| GO_ACTIN_CYTOSKELETON | 418 | 0.346708 | 1.6678512 | 0 | 0.171248 |
| GO_CYTOKINE_PRODUCTION_INVOLVED_IN_IMMUNE_RESPONSE | 16 | 0.598673 | 1.6645306 | 0.018828 | 0.174688 |
| GO_MYOSIN_II_COMPLEX | 21 | 0.573107 | 1.6617085 | 0.01232 | 0.177065 |
| GO_LYMPH_VESSEL_DEVELOPMENT | 20 | 0.571362 | 1.6609911 | 0.022822 | 0.176258 |
| GO_RESPONSE_TO_TYPE_I_INTERFERON | 60 | 0.44986 | 1.6593975 | 0 | 0.17677 |
| GO_HEPARAN_SULFATE_PROTEOGLYCAN_METABOLIC_PROCESS | 26 | 0.539557 | 1.6590319 | 0.004283 | 0.175399 |
| GO_POSITIVE_REGULATION_OF_INTERLEUKIN_12_PRODUCTION | 30 | 0.526344 | 1.6524485 | 0.008621 | 0.184015 |
| GO_ESTROUS_CYCLE | 15 | 0.622063 | 1.6520425 | 0.023305 | 0.182757 |
| GO_SYNAPTIC_MEMBRANE | 224 | 0.363729 | 1.6517246 | 0 | 0.181241 |
| GO_METANEPHRIC_NEPHRON_MORPHOGENESIS | 19 | 0.563806 | 1.6470181 | 0.015284 | 0.186848 |
| GO_POSITIVE_REGULATION_OF_LYMPHOCYTE_APOPTOTIC_PROCESS | 16 | 0.61179 | 1.6464939 | 0.010142 | 0.185879 |
| GO_SEGMENTATION | 80 | 0.420618 | 1.6430466 | 0.002193 | 0.189243 |
| GO_POSITIVE_REGULATION_OF_INTERFERON_ALPHA_PRODUCTION | 16 | 0.612407 | 1.638917 | 0.01232 | 0.194282 |
| GO_GASTRULATION | 136 | 0.382027 | 1.638239 | 0 | 0.193124 |
| GO_NEGATIVE_REGULATION_OF_PROTEIN_TYROSINE_KINASE_ACTIVITY | 19 | 0.583783 | 1.6378443 | 0.02 | 0.19197 |
| GO_PROTEIN_TYROSINE_PHOSPHATASE_ACTIVITY | 95 | 0.406929 | 1.6372725 | 0.002114 | 0.191093 |
| GO_POSITIVE_REGULATION_OF_TOLL_LIKE_RECEPTOR_SIGNALING_PATHWAY | 19 | 0.576959 | 1.6365978 | 0.019313 | 0.190313 |
| GO_NIK_NF_KAPPAB_SIGNALING | 80 | 0.430502 | 1.6365882 | 0.004566 | 0.18844 |
| GO_SOMITOGENESIS | 55 | 0.451994 | 1.6355056 | 0.002155 | 0.18819 |
| GO_POSITIVE_REGULATION_OF_COAGULATION | 19 | 0.567072 | 1.6346925 | 0.016194 | 0.187779 |
| GO_HEPARAN_SULFATE_PROTEOGLYCAN_BIOSYNTHETIC_PROCESS | 22 | 0.548124 | 1.6342103 | 0.022026 | 0.186796 |
| GO_NEGATIVE_REGULATION_OF_BIOMINERAL_TISSUE_DEVELOPMENT | 16 | 0.590617 | 1.6337774 | 0.011062 | 0.185789 |
| GO_CELL_JUNCTION_ORGANIZATION | 174 | 0.376566 | 1.6315997 | 0 | 0.187443 |
| GO_EPITHELIAL_CELL_MORPHOGENESIS | 38 | 0.489405 | 1.6315897 | 0.017621 | 0.185702 |
| GO_DEFENSE_RESPONSE_TO_VIRUS | 141 | 0.381963 | 1.6303574 | 0 | 0.18612 |
| GO_T_CELL_MEDIATED_IMMUNITY | 27 | 0.523368 | 1.6275979 | 0.01848 | 0.188544 |
| GO_CONTRACTILE_FIBER | 194 | 0.367383 | 1.6262848 | 0 | 0.188715 |
| GO_CARDIAC_MUSCLE_TISSUE_MORPHOGENESIS | 48 | 0.465255 | 1.621965 | 0.008869 | 0.194462 |
| GO_TRANSMEMBRANE_RECEPTOR_PROTEIN_TYROSINE_KINASE_ACTIVITY | 64 | 0.435954 | 1.6212817 | 0.004338 | 0.193905 |
| GO_CORTICAL_ACTIN_CYTOSKELETON | 57 | 0.44341 | 1.6191621 | 0.006237 | 0.195579 |
| GO_THREONINE_TYPE_PEPTIDASE_ACTIVITY | 19 | 0.568682 | 1.6190385 | 0.008529 | 0.194073 |
| GO_DOUBLE_STRANDED_RNA_BINDING | 58 | 0.448072 | 1.6186806 | 0.002222 | 0.192999 |
| GO_HEART_MORPHOGENESIS | 194 | 0.366411 | 1.6181067 | 0 | 0.192433 |
| GO_POSTSYNAPTIC_MEMBRANE | 175 | 0.370145 | 1.6148012 | 0.004587 | 0.19621 |
| GO_VENOUS_BLOOD_VESSEL_DEVELOPMENT | 15 | 0.613251 | 1.6132716 | 0.029106 | 0.197064 |
| GO_TRANSMEMBRANE_RECEPTOR_PROTEIN_KINASE_ACTIVITY | 80 | 0.416622 | 1.6126204 | 0.004348 | 0.196586 |
| GO_SOMITE_DEVELOPMENT | 70 | 0.429102 | 1.6114076 | 0.008368 | 0.197159 |
| GO_MESODERM_DEVELOPMENT | 105 | 0.389673 | 1.6109666 | 0 | 0.196153 |
| GO_STEM_CELL_DIFFERENTIATION | 171 | 0.367985 | 1.6093509 | 0 | 0.197213 |
| GO_REGULATION_OF_FIBROBLAST_GROWTH_FACTOR_RECEPTOR_SIGNALING_PATHWAY | 22 | 0.556417 | 1.6086736 | 0.015521 | 0.196759 |
| GO_REGULATION_OF_CYTOKINE_PRODUCTION_INVOLVED_IN_IMMUNE_RESPONSE | 50 | 0.450953 | 1.6068318 | 0.004415 | 0.198507 |
| GO_MYOFIBRIL_ASSEMBLY | 42 | 0.469084 | 1.6068095 | 0.006494 | 0.196955 |
| GO_TRANSMEMBRANE_RECEPTOR_PROTEIN_PHOSPHATASE_ACTIVITY | 17 | 0.570332 | 1.6053158 | 0.029167 | 0.197816 |
| GO_RECEPTOR_ACTIVATOR_ACTIVITY | 27 | 0.518005 | 1.6050489 | 0.015152 | 0.196697 |
| GO_CARDIAC_ATRIUM_DEVELOPMENT | 26 | 0.518811 | 1.6027104 | 0.014799 | 0.198811 |
| GO_POSITIVE_REGULATION_OF_T_CELL_MEDIATED_IMMUNITY | 31 | 0.499494 | 1.6016067 | 0.008403 | 0.199097 |
| GO_COLLAGEN_FIBRIL_ORGANIZATION | 36 | 0.484361 | 1.6001397 | 0.022883 | 0.199881 |
| GO_REGULATION_OF_CARDIAC_MUSCLE_CELL_ACTION_POTENTIAL | 18 | 0.572977 | 1.5998843 | 0.023861 | 0.198695 |
| GO_RUFFLE | 148 | 0.374489 | 1.5996336 | 0 | 0.197613 |
| GO_POSITIVE_REGULATION_OF_INTERLEUKIN_1_SECRETION | 18 | 0.561068 | 1.598441 | 0.023158 | 0.198096 |
| GO_INTEGRIN_MEDIATED_SIGNALING_PATHWAY | 81 | 0.405536 | 1.5983088 | 0.006711 | 0.196907 |
| GO_CANONICAL_WNT_SIGNALING_PATHWAY | 88 | 0.399874 | 1.5981237 | 0.002392 | 0.195728 |
| GO_REGULATION_OF_CELLULAR_AMINO_ACID_METABOLIC_PROCESS | 63 | 0.421888 | 1.5959741 | 0.002183 | 0.197726 |
| GO_TRANSFORMING_GROWTH_FACTOR_BETA_RECEPTOR_BINDING | 44 | 0.467619 | 1.5954078 | 0.006593 | 0.197371 |
| GO_MESENCHYME_DEVELOPMENT | 168 | 0.362918 | 1.5951909 | 0 | 0.196329 |
| GO_STRUCTURAL_CONSTITUENT_OF_CYTOSKELETON | 86 | 0.404397 | 1.5939196 | 0 | 0.196994 |
| GO_MESENCHYMAL_CELL_DIFFERENTIATION | 124 | 0.384386 | 1.59375 | 0.002342 | 0.195781 |
| GO_WNT_PROTEIN_BINDING | 28 | 0.503218 | 1.5911391 | 0.012739 | 0.19844 |
| GO_RESPIRATORY_GASEOUS_EXCHANGE | 46 | 0.455521 | 1.5907573 | 0.008565 | 0.197836 |
| GO_POSITIVE_REGULATION_OF_EPIDERMAL_CELL_DIFFERENTIATION | 17 | 0.572599 | 1.5897416 | 0.036638 | 0.19823 |
| GO_MEMBRANE_RAFT_ORGANIZATION | 15 | 0.596876 | 1.5897158 | 0.035941 | 0.196877 |
| GO_ACTIVATION_OF_PROTEIN_KINASE_B_ACTIVITY | 20 | 0.553699 | 1.5891747 | 0.024742 | 0.196294 |
| GO_MUSCLE_STRUCTURE_DEVELOPMENT | 390 | 0.332354 | 1.588573 | 0 | 0.19591 |
| GO_CALCIUM_ION_REGULATED_EXOCYTOSIS_OF_NEUROTRANSMITTER | 28 | 0.500702 | 1.5879833 | 0.021053 | 0.195493 |
| GO_CELL_ADHESION_MOLECULE_BINDING | 174 | 0.362292 | 1.5868082 | 0 | 0.196058 |
| GO_ENDOCARDIAL_CUSHION_MORPHOGENESIS | 22 | 0.547539 | 1.5866472 | 0.017316 | 0.195002 |
| GO_IONOTROPIC_GLUTAMATE_RECEPTOR_COMPLEX | 38 | 0.470168 | 1.5855589 | 0.011111 | 0.195518 |
| GO_RESPONSE_TO_ACETYLCHOLINE | 17 | 0.568083 | 1.582621 | 0.04814 | 0.198554 |
| GO_SMAD_PROTEIN_SIGNAL_TRANSDUCTION | 50 | 0.449929 | 1.5821847 | 0.01182 | 0.19793 |
| GO_REGULATION_OF_TOLL_LIKE_RECEPTOR_SIGNALING_PATHWAY | 44 | 0.455834 | 1.581979 | 0.015625 | 0.196934 |
| GO_ESTABLISHMENT_OR_MAINTENANCE_OF_BIPOLAR_CELL_POLARITY | 34 | 0.472523 | 1.5810858 | 0.016771 | 0.197048 |
| GO_CELL_MORPHOGENESIS_INVOLVED_IN_DIFFERENTIATION | 471 | 0.326479 | 1.5804043 | 0 | 0.19688 |
| GO_WNT_SIGNALING_PATHWAY | 328 | 0.337039 | 1.5802609 | 0 | 0.195874 |
| GO_POSITIVE_REGULATION_OF_CANONICAL_WNT_SIGNALING_PATHWAY | 112 | 0.38716 | 1.5788813 | 0 | 0.196767 |
| GO_LENS_MORPHOGENESIS_IN_CAMERA_TYPE_EYE | 16 | 0.579834 | 1.5771308 | 0.033755 | 0.198448 |
| GO_INNATE_IMMUNE_RESPONSE_ACTIVATING_CELL_SURFACE_RECEPTOR_SIGNALING_PATHWAY | 97 | 0.389135 | 1.5759063 | 0 | 0.199409 |
| GO_CELL_CORTEX | 214 | 0.349898 | 1.5750555 | 0.002358 | 0.199553 |
| GO_REGULATION_OF_KIDNEY_DEVELOPMENT | 52 | 0.440806 | 1.5739836 | 0.006912 | 0.200024 |
| GO_POSITIVE_REGULATION_OF_STEM_CELL_DIFFERENTIATION | 45 | 0.451635 | 1.572109 | 0.016842 | 0.201639 |
| GO_RESPONSE_TO_INTERFERON_ALPHA | 20 | 0.547365 | 1.5707313 | 0.013274 | 0.202581 |
| GO_SKELETAL_SYSTEM_DEVELOPMENT | 418 | 0.327502 | 1.5706503 | 0 | 0.201492 |
| GO_COPI_COATED_VESICLE_MEMBRANE | 16 | 0.575854 | 1.568714 | 0.029787 | 0.203473 |
| GO_CYCLIN_DEPENDENT_PROTEIN_KINASE_HOLOENZYME_COMPLEX | 29 | 0.494096 | 1.566668 | 0.023504 | 0.205721 |
| GO_POSITIVE_REGULATION_OF_INTERLEUKIN_6_PRODUCTION | 59 | 0.426534 | 1.5645738 | 0.008929 | 0.20786 |
| GO_EXTRACELLULAR_STRUCTURE_ORGANIZATION | 275 | 0.341561 | 1.5635016 | 0 | 0.208346 |
| GO_VASOCONSTRICTION | 24 | 0.508046 | 1.5605774 | 0.025641 | 0.212023 |
| GO_CELL_SURFACE_RECEPTOR_SIGNALING_PATHWAY_INVOLVED_IN_HEART_DEVELOPMENT | 15 | 0.592438 | 1.5598592 | 0.02439 | 0.212034 |
| GO_STEREOCILIUM_BUNDLE | 34 | 0.474982 | 1.5577265 | 0.01566 | 0.214296 |
| GO_POSITIVE_REGULATION_OF_BMP_SIGNALING_PATHWAY | 30 | 0.492887 | 1.5546181 | 0.022587 | 0.218606 |
| GO_ANTIGEN_PROCESSING_AND_PRESENTATION_OF_PEPTIDE_ANTIGEN | 161 | 0.355809 | 1.5540283 | 0 | 0.218276 |
| GO_ANCHORING_JUNCTION | 473 | 0.321265 | 1.5530276 | 0 | 0.218825 |
| GO_ESTABLISHMENT_OR_MAINTENANCE_OF_EPITHELIAL_CELL_APICAL_BASAL_POLARITY | 28 | 0.493308 | 1.5509638 | 0.020325 | 0.221187 |
| GO_NEGATIVE_REGULATION_OF_PEPTIDYL_TYROSINE_PHOSPHORYLATION | 37 | 0.465961 | 1.550236 | 0.029046 | 0.221283 |
| GO_POSITIVE_REGULATION_OF_CYTOKINESIS | 27 | 0.498303 | 1.5461407 | 0.024499 | 0.227268 |
| GO_IMMUNE_RESPONSE_REGULATING_CELL_SURFACE_RECEPTOR_SIGNALING_PATHWAY | 258 | 0.335943 | 1.5460004 | 0 | 0.226293 |
| GO_CELLULAR_EXTRAVASATION | 23 | 0.516044 | 1.545859 | 0.030172 | 0.225252 |
| GO_REGULATION_OF_DNA_DAMAGE_RESPONSE_SIGNAL_TRANSDUCTION_BY_P53_CLASS_MEDIATOR | 28 | 0.492902 | 1.5442442 | 0.02449 | 0.226696 |
| GO_WNT_SIGNALING_PATHWAY_CALCIUM_MODULATING_PATHWAY | 35 | 0.468132 | 1.5441778 | 0.027027 | 0.225557 |
| GO_NEPHRON_TUBULE_FORMATION | 16 | 0.569064 | 1.5440412 | 0.036957 | 0.224568 |
| GO_PROTEIN_PHOSPHATASE_1_BINDING | 17 | 0.556515 | 1.54278 | 0.038776 | 0.225576 |
| GO_PROTEIN_KINASE_B_SIGNALING | 32 | 0.479813 | 1.537306 | 0.015086 | 0.234428 |
| GO_REGULATION_OF_INTERLEUKIN_1_PRODUCTION | 49 | 0.430516 | 1.5365545 | 0.018519 | 0.234613 |
| GO_REGULATION_OF_ORGAN_MORPHOGENESIS | 225 | 0.339607 | 1.5335882 | 0 | 0.238995 |
| GO_CHONDROCYTE_DIFFERENTIATION | 56 | 0.414806 | 1.5335096 | 0.015086 | 0.237832 |
| GO_RHO_GUANYL_NUCLEOTIDE_EXCHANGE_FACTOR_ACTIVITY | 74 | 0.388562 | 1.5313516 | 0.002268 | 0.240577 |
| GO_CARDIAC_VENTRICLE_MORPHOGENESIS | 51 | 0.425981 | 1.531101 | 0.010823 | 0.239715 |
| GO_CARDIAC_MUSCLE_CELL_DIFFERENTIATION | 65 | 0.408676 | 1.5296313 | 0.009174 | 0.241156 |
| GO_POSITIVE_REGULATION_OF_EXTRINSIC_APOPTOTIC_SIGNALING_PATHWAY | 50 | 0.425432 | 1.5294168 | 0.011136 | 0.240317 |
| GO_SINGLE_ORGANISM_CELL_ADHESION | 394 | 0.319738 | 1.5273451 | 0 | 0.242687 |
| GO_MUSCLE_CELL_DIFFERENTIATION | 211 | 0.34209 | 1.5264083 | 0.002315 | 0.243052 |
| GO_RNA_POLYMERASE_II_ACTIVATING_TRANSCRIPTION_FACTOR_BINDING | 33 | 0.459164 | 1.5248299 | 0.021786 | 0.244614 |
| GO_FC_RECEPTOR_SIGNALING_PATHWAY | 171 | 0.347645 | 1.5219059 | 0.007026 | 0.248759 |

SIZE: Number of genes in the gene set after filtering out those genes not in the expression dataset, ES: Enrichment score for the gene set; that is, the degree to which this gene set is overrepresented at the top or bottom of the ranked list of genes in the expression dataset, NES: Normalized enrichment score; that is, the enrichment score for the gene set after it has been normalized across analyzed gene sets, NOM p-val: Nominal p value; that is, the statistical significance of the enrichment score. The nominal p value is not adjusted for gene set size or multiple hypothesis testing; therefore, it is of limited use in comparing gene sets, FDR q-value: False discovery rate; that is, the estimated probability that the normalized enrichment score represents a false positive finding.
